# Supplementary material for: Single-Equipment with Multiple-Application for an Automated Robot-Car Control System
Source: Sensors (Basel). 2019 Feb 6;19(3):662. doi: 10.3390/s19030662 (PMC6387148; doi:10.3390/s19030662)
Supplement: Supplementary file 1 [file sensors-19-00662-s001.pdf]

sensors

# Single-Equipment with Multiple-Application for an Automated Robot-Car Control System

**Saleem Ullah [1,†], Zain Mumtaz [1,†], Shuo Liu [2], Mohammad Abubaqr [1], Athar Mahboob [1,3] and Hamza Ahmad Madni [2,3,*]**

1 Department of Computer Science, Khwaja Fareed University of Engineering and Information Technology, Rahim Yar Khan 64200, Pakistan; saleem.ullah@kfueit.edu.pk (S.U.); zainmumtaz007@gmail.com (Z.M.); mohammadabubaqr@outlook.com (M.A); athar@kfueit.edu.pk (A.M.)
2 State Key Laboratory of Millimeter Waves, Department of Radio Engineering, Southeast University, Nanjing 210096, China; liushuo.china@seu.edu.cn
3 Department of Computer Engineering, Khwaja Fareed University of Engineering and Information Technology, Rahim Yar Khan 64200, Pakistan
* Correspondence: 101101770@seu.edu.cn; Tel.: +86-1782-6500-615 or +92-313-4820-856
† These authors contributed equally to this work.

## 1. Supplementary Material

### *1.1. Hand-Gestures Recognition*

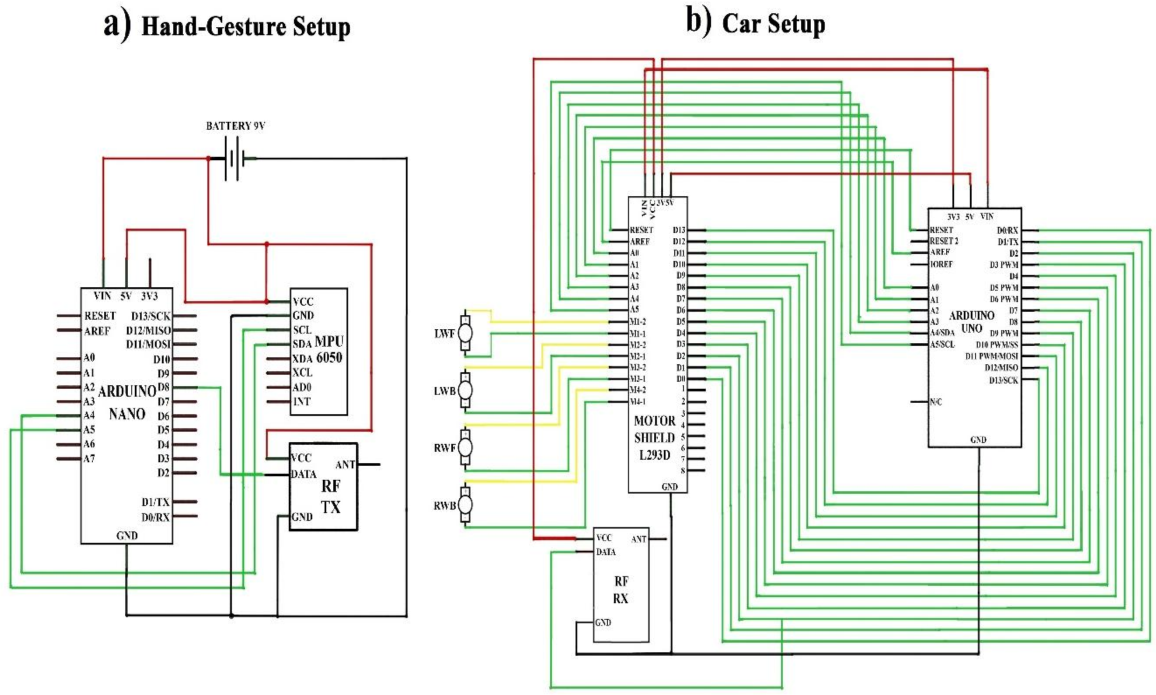


**Figure S1.** The circuit design of car controlling system with Arduino using hand gestures recognition. (**a**) Shows the schematic of hand-gesture setup. (**b**) Display the schematic design of car setup.

Figure S1 shows the circuit design of Hand gestures which control the robotic car using Arduino. In this scenario, the robotic car will move in the same direction as the direction of hand rotation. There are two schematic diagrams for the hand-gesture; Figure S1a is for the hand-gesture setup in which one Arduino Nano, one MPU-6050 accelerometer and one RF transmitter were used and Figure S1b is for the car setup in which one L293D motor module, one Arduino Uno and one RF-receiver were used.

All the explanation of Hand-Gestures Recognition System has been described in the Figure 2 of main manuscript and the code is written below:

1.1.1. Code for Hand-Gesture Setup

```
//Connect the RF TX data pin to Arduino pin 8
int xPin=4;//Connect x pin of MPU 6050 to pin A4
int yPin=5;//Connect y pin of MPU 6050 to pin A5

#include <VirtualWire.h>
void setup()
{
  vw_setup(2000);
  pinMode(ledPin,OUTPUT);
  Serial.begin(9600);
}
void loop()
{
  int xval=analogRead(xPin);
  int yval=analogRead(yPin);
  Serial.print("xval=");
  Serial.println(xval);
  Serial.print("yval=");
  Serial.println(yval);
  delay(1000);
  Serial.print("\n");
  if ((xval>330 && xval<340) && (yval>340 && yval<350)) // Brake
  {
    digitalWrite(ledPin,LOW);
    send("s");
  }
  else
  {
    if ((xval>325 && xval<345) && (yval>380 && yval<410)) //forward movement
    {
      digitalWrite(ledPin,HIGH);
      send("f");
    }
    if ((xval>315 && xval<345) && (yval>250 && yval<275)) //backward movement
    {
      digitalWrite(ledPin,HIGH);
      send("a");
    }
    if ((xval>385 && xval<415) && (yval>315 && yval<350)) //left movement
    {
      digitalWrite(ledPin,HIGH);
      send("l");
     }
     if ((xval>255 && xval<275) && (yval>320 && yval<345))//right movement
    {
      digitalWrite(ledPin,HIGH);
      send("r");
    }
  }
  delay(1000);
}

void send(char *message)
{
  vw_send((uint8_t *)message, strlen(message));
  vw_wait_tx();
```

```
}
```

1.1.2. Code for Car Setup

```
//Connect the RF RX data pin to Arduino pin 2
#include <AFMotor.h> //including library
#define TPin 6
#define EPin 5
#include <VirtualWire.h>
byte message[VW_MAX_MESSAGE_LEN];
byte messageLength = VW_MAX_MESSAGE_LEN;
int lm=12;
int lmr=8;
int rm=10;
int rmr=7;
int ledPin=13;
void distance()
{
  long duration, distance;
  digitalWrite(TPin, LOW);
  delayMicroseconds(2);
  digitalWrite(TPin, HIGH);

  delayMicroseconds(10);
  digitalWrite(TPin, LOW);
  duration = pulseIn(EPin, HIGH);
  distance = (duration/2) / 29.1;
  if (distance < 20)/*if there's an obstacle 20 centimers, ahead, do the following: */ {
   Serial.println ("Close Obstacle detected!" );
Serial.println ("Obstacle Details:");
Serial.print ("Distance From Robot is " );
Serial.print ( distance);
Serial.print ( " CM!");
if(Serial.available())
{
  t = Serial.read();
  Serial.println(t);
}
if(distance<25)
{
void setup()
{
 Serial.begin(9600);
 pinMode(ledPin,OUTPUT);
 pinMode(lm,OUTPUT);
 pinMode(lmr,OUTPUT);
 pinMode(rm,OUTPUT);
 pinMode(rmr,OUTPUT);
 vw_setup(2000);
 vw_rx_start();
}
void loop()
{
    uint8_t buf[VW_MAX_MESSAGE_LEN];
    uint8_t buflen = VW_MAX_MESSAGE_LEN;

    if (vw_get_message(buf, &buflen))
    {
   int i;
```

```
Serial.print("Got: ");//debugging

     for (i = 0; i < buflen; i++)
     {
          // Serial.print(buf[i],HEX);
           //Serial.print(' ');// debugging

                if (buf[i]==0x73)//Stationary
                {
                   digitalWrite(lm,LOW);
                   digitalWrite(lmr,LOW);
                   digitalWrite(rm,LOW);
                   digitalWrite(rmr,LOW);

                   digitalWrite(ledPin,LOW);
                }
                else
                {
                   if(buf[i]==0x66)//Forward
                   {
                      digitalWrite(lm,LOW);
                      digitalWrite(lmr,HIGH);
                      digitalWrite(rm,HIGH);
                      digitalWrite(rmr,LOW);

                      digitalWrite(ledPin,HIGH);
                   }

                   if (buf[i]==0x61)//Backward
                   {
                      digitalWrite(lm,HIGH);
                      digitalWrite(lmr,LOW);
                      digitalWrite(rm,LOW);
                      digitalWrite(rmr,HIGH);

                      digitalWrite(ledPin,HIGH);
                }

                   if (buf[i]==0x6C)//Left
                   {
                      digitalWrite(lm,LOW);
                      digitalWrite(lmr,LOW);
                      digitalWrite(rm,HIGH);
                      digitalWrite(rmr,LOW);
                      digitalWrite(ledPin,HIGH);
                   }

                   if (buf[i]==0x72)//Right
                   {
                      digitalWrite(lm,LOW);
                      digitalWrite(lmr,HIGH);
                      digitalWrite(rm,LOW);
                      digitalWrite(rmr,LOW);
                      digitalWrite(ledPin,HIGH);
                   }
                 }
        }
        //Serial.print("\n");
             }
```

```
        //delay(1000)
}
}
else
{
            digitalWrite(lm,LOW);
            digitalWrite(lmr,LOW);
            digitalWrite(rm,LOW);
            digitalWrite(rmr,LOW);
            digitalWrite(ledPin,LOW);
}
}
```

### *2.1. Mobile Application System*

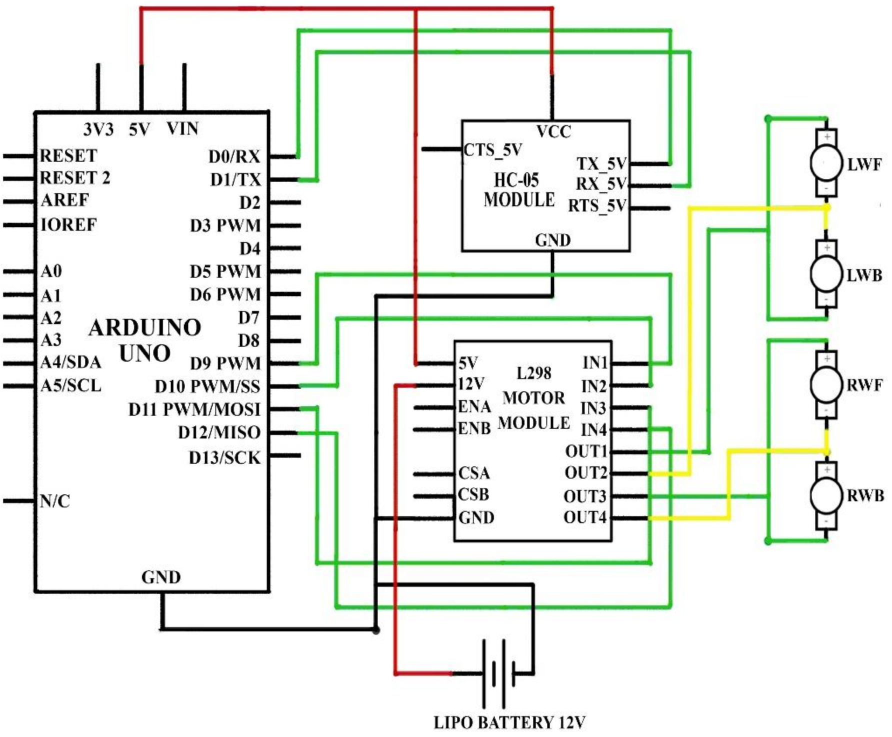


**Figure S2.** The circuit design of mobile application to control car.

Figure S2 shows circuit design of controlling the robot car based on Android mobile application. In this task, an Arduino UNO, one L298 motor module, one HC-05 Bluetooth module, one 12V battery and four DC motors are used.

All the explanation of Object Independent Automation System has been described in the Figure 6 of main manuscript and the code is written below:

```
#include <AFMotor.h>
#define TPin 7
#define EPin 8
char t;
void setup()
{
pinMode(13,OUTPUT);    //left motors forward
pinMode(12,OUTPUT);    //left motors reverse
pinMode(11,OUTPUT);    //right motors forward
pinMode(10,OUTPUT);    //right motors reverse
Serial.begin(9600);
```

```
 pinMode(TPin, OUTPUT);
  pinMode(EPin, INPUT);
}
void loop()
{
  long duration, distance;
  digitalWrite(TPin, LOW);
  delayMicroseconds(2);
  digitalWrite(TPin, HIGH);
  delayMicroseconds(10);
  digitalWrite(EPin, LOW);
  duration = pulseIn(EPin, HIGH);
  distance = (duration/2) / 29.1;
  if (distance < 20)/*if there's an obstacle 20 centimers, ahead, do the following: */ {
   Serial.println ("Close Obstacle detected!" );
Serial.println ("Obstacle Details:");
Serial.print ("Distance From Robot is " );
Serial.print ( distance);
Serial.print ( " CM!");// print out the distance in centimeters.
if(Serial.available())
{
  t = Serial.read();
  Serial.println(t);
}
if(t == '1')
{
if(distance<13)
{
  digitalWrite(13,LOW);
  digitalWrite(12,LOW);
  digitalWrite(11,LOW);
  digitalWrite(10,LOW);//move forward(all motors rotate in forward direction)
}
else
{
  digitalWrite(13,HIGH);
  digitalWrite(12,LOW);
  digitalWrite(11,HIGH);
  digitalWrite(10,LOW);
}
}
else if(t == '2')
{
  //move reverse (all motors rotate in reverse direction)
if(distance<13)
{
  digitalWrite(13,LOW);
  digitalWrite(12,LOW);
  digitalWrite(11,LOW);
  digitalWrite(10,LOW);//move forward(all motors rotate in forward direction)
}
else
{
  digitalWrite(13,LOW);
  digitalWrite(12,HIGH);
  digitalWrite(11,LOW);
  digitalWrite(10,HIGH);
}
}
```

```
else if(t == '3')
{        //turn right (left side motors rotate in forward direction, right side motors doesn't rotate)
 if(distance<13)
{
  digitalWrite(13,LOW);
  digitalWrite(12,LOW);
  digitalWrite(11,LOW);
  digitalWrite(10,LOW);//move forward(all motors rotate in forward direction)
}
else
{
  digitalWrite(13,LOW);
  digitalWrite(12,LOW);
  digitalWrite(11,HIGH);
  digitalWrite(10,LOW);
}
}
else if(t == '4')
{
  if(distance<13)
{
  digitalWrite(13,LOW);
  digitalWrite(12,LOW);
  digitalWrite(11,LOW);
  digitalWrite(10,LOW);//move forward(all motors rotate in forward direction)
}
else
{//turn left (right side motors rotate in forward direction, left side motors doesn't rotate)
  digitalWrite(13,HIGH);
  digitalWrite(12,LOW);
  digitalWrite(11,LOW);
  digitalWrite(10,LOW);
}
}
else if(t == '5')
{
  if(distance<13)
{
  digitalWrite(13,LOW);
  digitalWrite(12,LOW);
  digitalWrite(11,LOW);
  digitalWrite(10,LOW);//move forward(all motors rotate in forward direction)
}
else
{
  digitalWrite(13,LOW);
  digitalWrite(12,LOW);
  digitalWrite(11,LOW);
  digitalWrite(10,LOW);
}
}
        delay(100);
```

© 2019 by the authors. Submitted for possible open access publication under the terms and conditions of the Creative Commons Attribution (CC BY) license (http://creativecommons.org/licenses/by/4.0/).
